# Genome-wide association study identifies *GAK* and *KLF12* associated with curve severity of adolescent idiopathic scoliosis

**DOI:** 10.7717/peerj.20638

**Published:** 2026-01-19

**Authors:** Zhicheng Dai, Zhichong Wu, Leilei Xu, Zhenhua Feng, Yong Qiu, Zezhang Zhu

**Affiliations:** 1 Division of Spine Surgery, Department of Orthopedic Surgery, Nanjing Drum Tower Hospital Clinical College of Jiangsu University, Nanjing, China; 2 Division of Spine Surgery, Department of Orthopedic Surgery, Nanjing Drum Tower Hospital, Affiliated Hospital of Medical School, Nanjing University, Nanjing, China

**Keywords:** Adolescent idiopathic scoliosis, GWAS, SNP, Curve severity

## Abstract

**Background:**

Genetic factors have been increasingly recognized as important contributors to the development and progression of adolescent idiopathic scoliosis (AIS). However, the genetic basis underlying AIS curve severity remains largely unclear. The objective of this study is to identify novel genetic variants associated with curve severity in AIS through a genome-wide association study (GWAS).

**Methods:**

In the discovery stage, 620 female AIS patients were enrolled, including 323 with severe curves (> 40°) and 297 with mild curves (< 30°). Top single nucleotide polymorphisms (SNPs) from each locus were selected for replication in an independent cohort of 634 severe and 546 mild cases. Associations between gene expression and Cobb angle were evaluated using Spearman correlation, while correlations with myofiber–related genes were analyzed using Pearson correlation.

**Results:**

Fifteen novel SNPs showed potential association with AIS curve severity in the discovery stage (*P* < 1 × 10^−4^). Six lead SNPs were selected for replication, including rs2061846 (*GAK*), rs12200301 (*DST*), rs10820637 (*SMC2/NIPSNAP3A*), rs7330031 (*KLF12*), rs2469472 (*ST8SIA5-DT*), and rs738650 (*SEZ6L*). Among these, rs2061846 and rs7330031 were successfully replicated. For rs2061846 in *GAK*, the frequency of the G allele was significantly higher in the severe group (*P* = 0.001; OR = 1.32). For rs7330031 in *KLF12*, the C allele was significantly more frequent in the severe group than in the mild group (*P* = 0.001; OR = 1.46). Moreover, *KLF12* mRNA expression in the paraspinal muscle of AIS patients was negatively correlated with Cobb angle and associated with muscle fiber–specific gene expression.

**Conclusions:**

This study identified *GAK* and *KLF12* as novel susceptibility genes associated with AIS curve severity, providing new insights into the genetic basis of curve progression. These findings may contribute to improved risk stratification and personalized management in AIS.

## Introduction

Adolescent idiopathic scoliosis (AIS) is a complex three-dimensional spinal deformity characterized by lateral curvature and vertebral rotation in the absence of congenital spinal anomalies (Cheng et al., 2015). Despite numerous studies, the pathogenesis of AIS remains unclear (Weinstein et al., 2008). Natural history of AIS indicated that spinal curves may progress substantially before patients reach skeletal maturity (Cheung et al., 2018; Yu et al., 2023). For severe cases with curve exceeding 50 degrees, surgical intervention is commonly recommended to prevent further progression and associated complications (Jinnah et al., 2024; Weinstein et al., 2013). Given the clinical burden and long-term impact of severe AIS, early identification of individuals at high risk for curve progression is critically important.

Several clinical factors have been reported to be associated with AIS progression, including initial curve magnitude, bone mineral density, and skeletal maturity (Shibata et al., 2025; Wong, Cheung & Cheung, 2022; Zhang et al., 2020). In recent years, growing attention has been paid to the molecular and genetic mechanisms underlying the curve progression in AIS. Previous studies have suggested possible roles for melatonin signaling, elevated inflammatory markers such as YKL-40 (Nada et al., 2019), and epigenetic modifications in modulating curve progression (Carry et al., 2021; Meng et al., 2018; Wu et al., 2025). From a genetic perspective, the *ScoliScore™* test was developed to predict curve progression risk based on common genetic variants, but its predictive value could not be replicated in non-European populations (Roye et al., 2015; Tang et al., 2015; Ward et al., 2010; Xu et al., 2016). These findings underscore the need for broader, population-specific investigations into the genetic markers for predicting AIS progression.

Over the past decade, genome-wide association studies (GWASs) have identified multiple AIS susceptibility loci, including *LBX1, GPR126, BNC2, PAX3, and TNIK* (Kou et al., 2013; Ogura et al., 2015; Takahashi et al., 2011; Zhu et al., 2015; Zhu et al., 2017). However, relatively few studies have focused on the genetic contribution to curve severity or progression. Miyake et al. (2013) identified a susceptibility locus for severe AIS on chromosome 17q24.3 through GWAS in a cohort of Japanese and Chinese population. Ogura et al. (2017) reported that a functional variant in *MIR4300HG* is associated with progression of adolescent idiopathic scoliosis. Nevertheless, the overall genetic architecture underlying progression to severe scoliosis remains largely unexplored. Therefore, there is a pressing need for more large-scale genome-wide association studies (GWAS) to identify susceptibility loci associated with AIS severity.

Previously, we conducted the first GWAS of AIS in a Chinese population and identified several novel genetic loci associated with disease susceptibility (Zhu et al., 2015). In the present study, we extended this work by focusing on genetic variants associated with curve severity. By reanalyzing our GWAS dataset and replication in an independent cohort, we identified two novel susceptibility loci, *KLF12* and *GAK*, which may be potentially involved in the progression of AIS. Our findings provide new insight into the genetic basis of curve severity and may contribute to future efforts in risk prediction and targeted intervention for AIS.

## Materials & Methods

### Subjects

This study was conducted under the approval of the Institutional Review Board of Nanjing Drum Tower Hospital (Approval No. 2019-066-01). All participants were informed of the study’s purpose and provided written consent. The current association analysis was composed of the initial genome-wide discovery stage followed by an independent replication stage. Patients who received treatment for scoliosis in our center between 2000 and 2017 were reviewed. The following inclusion criteria were used: (1) female patients diagnosed as AIS through clinical and radiologic examinations; (2) with major thoracic curve; (3) with Cobb angle less than 30 degrees or more than 40 degrees at skeletal maturity. Overall, 620 female AIS patients were enrolled in the discovery stage of GWAS. 323 patients with curve more than 40 degrees were assigned to the severe group and the other 297 patients with curve less than 30 degrees were assigned to the mild group. For the replication stage, an independent cohort of 634 patients with Cobb angle more than 40 degrees and 546 patients with Cobb angle less than 30 degrees were then included. All participants included in this study were from ethnic Han Chinese populations. Informed consent was obtained from the participants or from their guardians.

### Genotyping and quality control

Genomic DNA was isolated from peripheral blood leukocytes using a commercial DNA extraction kit (Qiagen, Hilden, Germany). In the discovery phase, genotyping was conducted with the Affymetrix Genome-Wide Human SNP Array 6.0 platform, adhering to the manufacturer’s protocol. Quality control of the raw genotyping data in the discovery phase was performed using PLINK (v1.90), as we previously described (Zhu et al., 2015). Single nucleotide polymorphisms (SNPs) that did not meet the following criteria were excluded: (1) call rates <95%; (2) minor allele frequency <0.05; (3) significant deviation from Hardy-Weinberg equilibrium (*P* <  1  × 10^−^^5^); and (4) located on non-autosomal chromosomes. For sample quality control, cryptic relatedness was assessed using an identity-by-state method, and individuals with second-degree or closer relationships were excluded. Additionally, individuals with more than 10% missing genotype data were also removed. To detect population outliers and stratification, we used principal component analysis (PCA) implemented in the software package EIGENSTRAT.

### Selection of SNPs for the replication

SNPs surpassing a threshold of *P* <  1.0  × 10^−4^ in the discovery stage were selected for further replication. The SNP genotyping in the replication stage was performed using TaqMan SNP Genotyping was conducted using an ABI Step-One-Plus sequence detection system (Applied Biosystems, Foster City, CA). To assess reproducibility, 5% of the samples were randomly chosen as blind duplicates, which demonstrated a 100% concordance. DNA samples with more than 10% missing data were excluded from further analysis.

### Genotype imputation

Genotype imputation within a 400kb window surrounding the two replicated loci (rs2061846 and rs7330031) was conducted using MaCH-Admix software (Liu et al., 2012). The reference data for linkage disequilibrium (LD) and haplotypes were sourced from the 1,000 Genomes Project, specifically from the phased CHB and CHS datasets (March 2012 release). Variants with imputation quality R^2^ < 0.30 or minor allele frequency <0.10 were excluded. Logistic regression models assuming an additive genetic effect were used to evaluate the association between imputed variants and AIS curve severity. Regional association plots were generated using LocusZoom (Pruim et al., 2010).

### Functional annotation

The regulatory properties of the novel susceptible signals were analyzed using the Chromatin state segmentation in LCL data generated by the ENCODE project. HaploReg Version 4.2 (Ward & Kellis, 2016) and RegulomeDB Version 2.2 (Boyle et al., 2012) were used to explore the annotations of the susceptible loci on haplotype blocks and to determine whether the variants are located in putative transcription factor binding sites or enhancer elements.

### Tissue collection and gene expression analysis

Paraspinal muscle samples from the proximal vertebra region were obtained during corrective surgery from 24 AIS patients for analysis of susceptible gene expression. The collected tissue was immediately flash-frozen in liquid nitrogen and stored at −80 °C for subsequent analysis. Total RNA was extracted with Trizol (Thermo Fisher Scientific, Waltham, MA, USA) according to the manufacturer’s protocol. A total of one µg RNA from each sample was reverse-transcripted to cDNA with the HiScript III All-in-one RT SuperMix Perfect for qPCR (Vazyme Biotech, Shanghai, China), followed by qPCR with the HiScript III All-in-one RT SuperMix Perfect for qPCR (Vazyme Biotech, Shanghai, China). Target gene expression values were calculated using the 2^−ΔCt^ method and normalized to *GAPDH* expression (Liu et al., 2012). Specific primers were listed in Table S1.

### Statistical analysis

PLINK 1.90 was used for general statistical analysis. The Cochran-Armitage trend test was used to calculate the association of each SNP with curve severity in the discovery stage. For the replication stage, the Chi-square test was used to compare the frequencies of genotype and allele between the mild and the severe group. OR values and 95% CIs were calculated from a 2 ×2 allele frequency table. The Manhattan plot was generated using Haploview version 4.2 (Barrett et al., 2004). A quantile–quantile plot generated with R (v2.6) was used to evaluate the potential impact of population stratification (Turner, 2018). Gene expression correlation analysis was performed with Pearson’s correlation test, or Spearman’s correlation test. Statistical significance was set at a *P* value of 0.05.

## Results

### Demographic characteristics of the subjects

Clinical characteristics of the subjects in the discovery and replication stage are summarized in Table 1. The mean age of the patients was 17.8 ± 1.9 years, ranging from 16.5 years to 22 years. All patients were followed up until skeletal maturity with a Risser grade of 5. The average curve magnitude was 41.5 ± 16.7 degrees, with a range of 20 to 72 degrees. For subjects included in the gene expression analysis, the mean Cobb angle were 55.5 ± 6.5 degrees, ranging from 45 to 72 degrees.

**Table 1:** Baseline characteristics of the subjects in discovery stage.

	Mean	Range
Age (years)	17.8 ± 1.9	16.5–22
Curve magnitude (degrees)	41.5 ± 16.7	20–72
Year post-menarche (years)	5.5 ± 1.3	4–7
Body mass index (kg/m^2^)	17.5 ± 2.3	16.1–22.3

### Association analysis and identification of severity-related loci

As shown in the Q-Q plot (Fig. S1A), the genome inflation factor (*λ*) was 1.02, indicating a lower possibility of population stratification. PCA analysis indicated that all subjects were genetically well-matched with minimal evidence of population stratification (Fig. S1B). After quality control, 606,254 SNPs were retained in the severe *versus* mild AIS association analysis.

**Figure 1:**
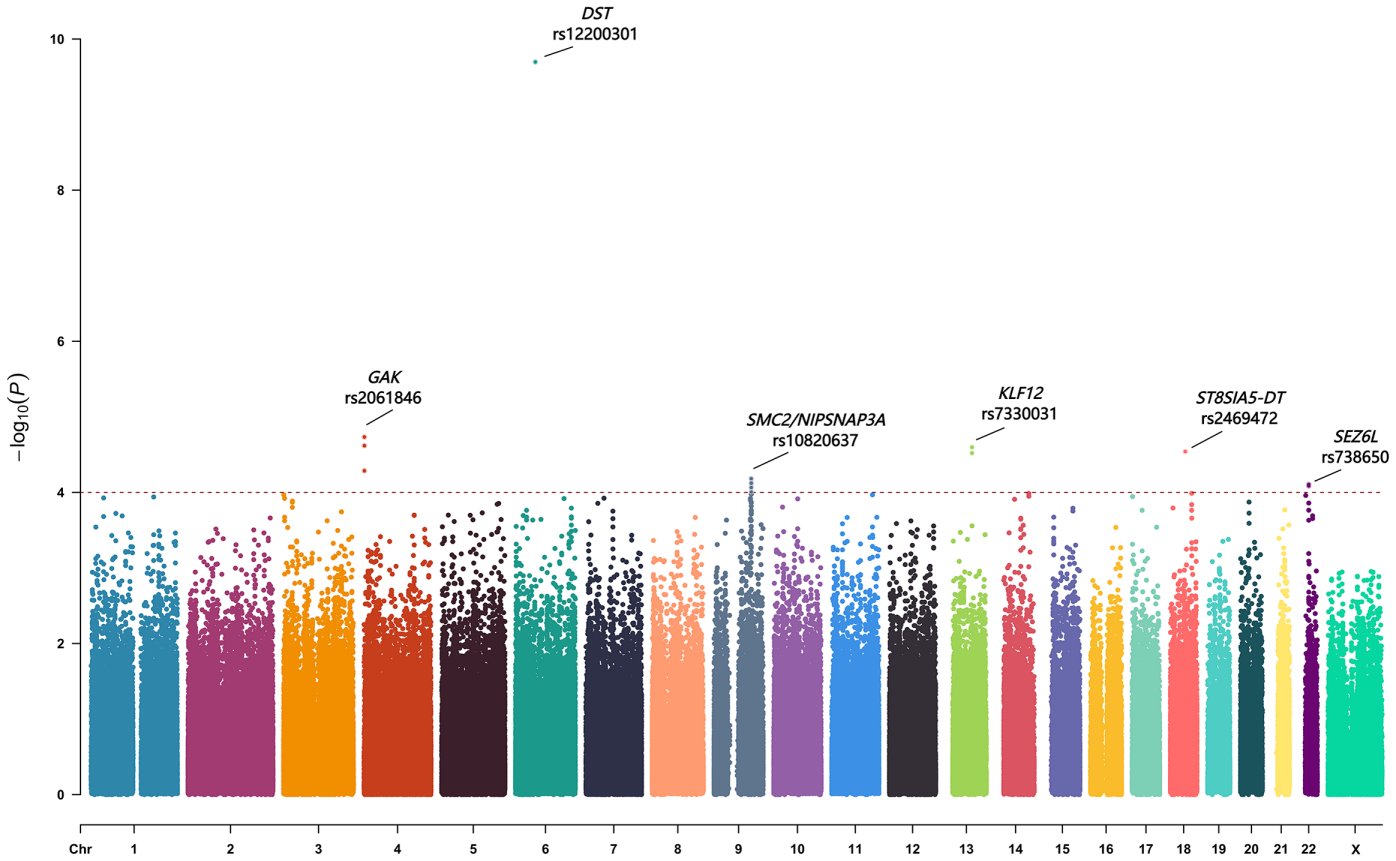
The Manhattan plot showing negative log10-transformed *P* values in the discovery stage. The dashed line represents the threshold of the suggestive whole-genome significance (*P* < 1.0 ×10^−4^). Fifteen SNPs were found to have a *p* value of less than 1.0 ×10^−4^. Six lead SNPs were finally included in the replication stage.

Fifteen SNPs showed a *p*-value of less than 1.0  × 10^−4^ (Table 2). These SNPs located within or near several genes, including *GAK, DST, SMC2/ NIPSNAP3A, KLF12, ST8SIA5-DT* and *SEZ6L*. We also evaluated previously reported AIS progression–associated variants identified in earlier GWASs (Miyake et al., 2013; Nada et al., 2019; Ogura et al., 2015; Ogura et al., 2017), but none reached suggestive significance in our cohort (Table S2). The top-associated SNPs from each locus (rs2061846, rs12200301, rs10820637, rs7330031, rs2469472, and rs738650) were selected for replication in an independent cohort (Fig. 1).

**Table 2:** Summary of the SNPs with suggestive significance in the discovery stage.

SNP	CHR	Genes	MA	MAF†		P†	OR	95% CI
				Severe (*n* = 323)	Mild (*n* = 297)			
**rs2061846**	**4**	***GAK***	**G**	**0.321**	**0.211**	**1.85 × 10** ^−5^	**1.769**	**(1.36–2.30)**
rs4690336	4	*GAK*	A	0.348	0.236	2.41 × 10^−5^	1.727	(1.34–2.22)
rs17165130	4	GAK	A	0.348	0.242	5.18 × 10-5	1.670	(1.30–2.14)
**rs12200301**	**6**	***DST***	**G**	**0.546**	**0.359**	**2.01 × 10** ^−10^	**2.142**	**(1.69–2.71)**
**rs10820637**	**9**	***SMC2/*** ***NIPSNAP3A***	**A**	**0.303**	**0.201**	**6.59 × 10** ^−5^	**1.721**	**(1.32–2.25)**
rs9644966	9	*SMC2/NIPSNAP3A*	G	0.308	0.207	7.50 × 10^−5^	1.706	(1.31–2.23)
rs10820640	9	*SMC2/NIPSNAP3A*	T	0.313	0.213	8.62 × 10^−5^	1.689	(1.29–2.19)
rs2417517	9	*SMC2/NIPSNAP3A*	T	0.312	0.213	9.89 × 10^−5^	1.681	(1.29–2.19)
rs1907678	9	*SMC2/NIPSNAP3A*	G	0.312	0.213	9.89 × 10^−5^	1.681	(1.29–2.19)
rs10991268	9	*SMC2/NIPSNAP3A*	A	0.264	0.169	7.61 × 10^−5^	1.762	(1.33–2.34)
**rs7330031**	**13**	***KLF12***	**C**	**0.150**	**0.072**	**2.53 × 10** ^−5^	**2.262**	**(1.54–3.33)**
rs879800	13	*KLF12*	A	0.162	0.078	3.02 × 10^−5^	2.259	(1.52–3.28)
**rs2469472**	**18**	***ST8SIA5-DT***	**T**	**0.278**	**0.175**	**2.88 × 10** ^−5^	**1.806**	**(1.37–2.39)**
**rs738650**	**22**	***SEZ6L***	**T**	**0.510**	**0.396**	**7.84 × 10** ^−5^	**1.588**	**(1.26–1.99)**
rs2847316	22	*SEZ6L*	G	0.525	0.410	8.22 × 10^−5^	1.587	(1.26–1.99)

**Notes.**

CHR chromosome MA minor allele MAF minor allele frequency OR odds ratio for the minor allele 95% CI 95% confidence intervals; The top-associated SNP from each locus is highlighted in bold

### Replication of severity-associated loci and locus-specific imputation

Replication analysis (Table 3) validated the association of two SNPs: rs2061846 in *GAK* and rs7330031 in *KLF12*. For rs2061846, the frequency of allele G was significantly higher in the severe AIS group compared to the mild group (35.3% *vs.* 29.3%, *P* = 0.001), with an odds ratio (OR) of 1.32 and a 95% confidence interval (CI) [1.11–1.57]. For rs7330031, allele C was more frequent in severe cases than in mild cases (16.9% *vs.* 12.2%, *p* = 0.001), with an OR of 1.46 and a 95% CI [1.16–1.85]. The remaining four SNPs did not show significant differences in allele or genotype distribution between groups.

**Table 3:** Replication results of the top-associated SNPs.

SNP	Genotype			*P*	Allele		*P*	Odds ratio (95% CI)
**rs2061846**	GG	GA	AA	**0.006**	G	A	**0.001**	1.32 (1.11–1.57)
Severe group (*n* = 634)	79 (12.5%)	290 (45.7%)	265 (41.8%)		448 (35.3%)	820 (64.7%)		
Mild group (*n* = 546)	52 (9.5%)	216 (39.6%)	278 (50.9%)		320 (29.3%)	772 (70.7%)		
rs12200301	GG	GA	AA	0.42	G	A	0.21	1.11 (0.96–1.31)
Severe group (*n* = 634)	140 (22.1%)	312 (49.2%)	182 (28.7%)		592 (46.7%)	676 (53.3%)		
Mild group (*n* = 546)	110 (20.1%)	261 (47.8%)	175 (32.1%)		481 (44.0%)	611 (56.0%)		
**rs10820637**	AA	AG	GG	0.56	A	G	0.28	1.10 (0.92–1.32)
Severe group (*n* = 634)	46 (7.3%)	271 (42.7%)	317 (50.0%)		363 (28.6%)	905 (71.4%)		
Mild group (*n* = 546)	41 (7.5%)	209 (38.3%)	296 (54.2%)		291 (26.6%)	801 (73.4%)		
**rs7330031**	CC	CA	AA	**0.002**	C	A	**0.001**	1.46 (1.16–1.85)
Severe group (*n* = 634)	21 (3.3%)	172 (27.1%)	441 (69.6%)		214 (16.9%)	1,054 (83.1%)		
Mild group (*n* = 546)	16 (2.9%)	101 (18.5%)	429 (78.6%)		133 (12.2%)	959 (87.8%)		
rs2469472	TT	TC	CC	0.53	T	C	0.31	1.11 (0.92–1.34)
Severe group (*n* = 634)	32 (5.0%)	248 (39.1%)	354 (55.8%)		312 (24.6%)	956 (75.4%)		
Mild group (*n* = 546)	22 (4.0%)	204 (37.4%)	320 (58.6%)		248 (22.7%)	844 (77.3%)		
rs738650	TT	TA	AA	0.72	T	A	0.51	1.06 (0.89–1.24)
Severe group (*n* = 634)	114 (18.0%)	336 (53.0%)	184 (29.0%)		564 (44.5%)	704 (55.5%)		
Mild group (*n* = 546)	109 (20.0%)	253 (46.3%)	184 (33.7%)		471 (43.1%)	621 (56.9%)		

**Note.**

Loci that remained significantly associated in the validation analysis are indicated in bold.

**Figure 2:**
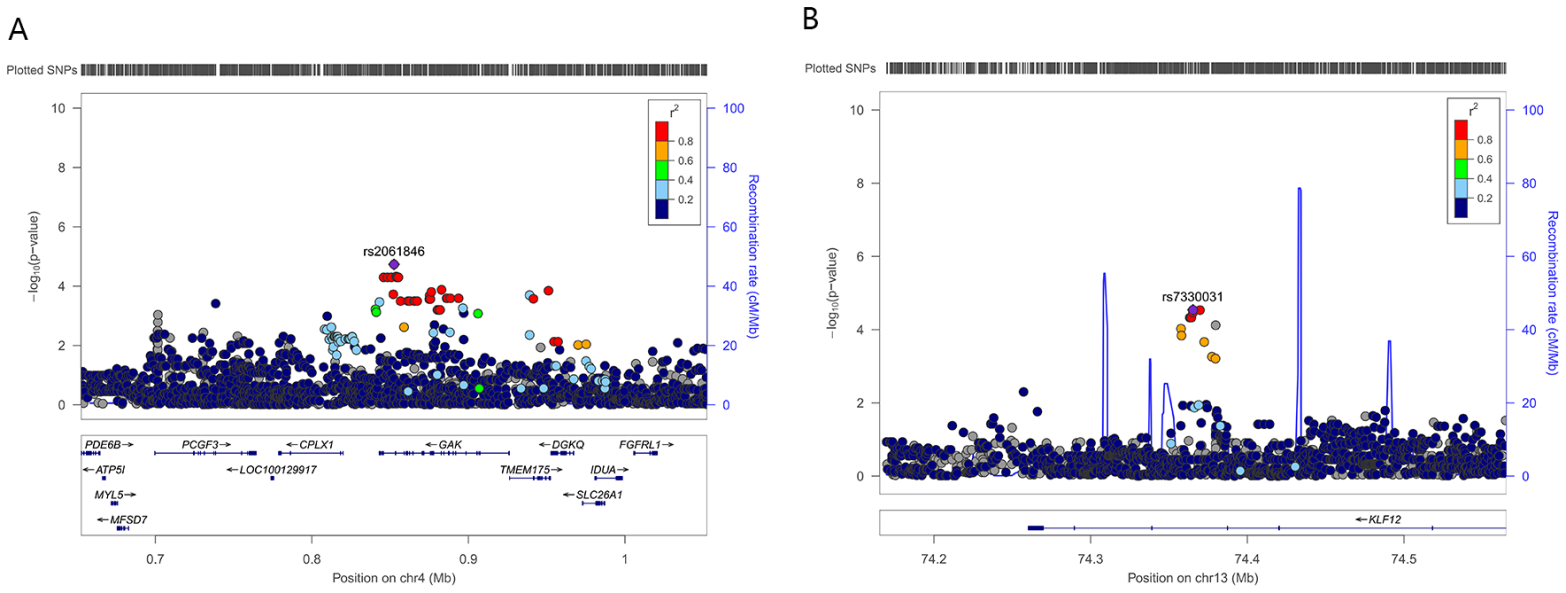
Regional association plots of two novel susceptibility loci for AIS severity. Each plot shows −log 10 (*P* value) for SNPs in the specific region. The genotyped SNP with the strongest association signal in each locus is indicated by a purple diamond, and the other SNPs are colored according to the *r*^2^ values with the proxy SNP. The genes within the regions of interest are annotated with the direction of transcription represented by arrows. (A) 4p16.3, (B) 13q22.1.

To further refine the regional association signal, locus-specific imputation was performed across ±400 kb of rs2061846 and rs7330031 (Fig. 2). The association patterns of imputed variants closely mirrored those of the directly genotyped SNPs, and no imputed variant exceeded the statistical significance of the two lead SNPs. These results indicate that rs2061846 and rs7330031 represent the primary association signals within their respective LD blocks.

### Relationship between expression levels of susceptibility genes and phenotypes in AIS

The two novel susceptibility loci, rs2061846 and rs7330031, are located within intronic regions of the *GAK* and *KLF12* genes, respectively. To investigate their potential roles in AIS pathogenesis, we examined the mRNA expression levels of *GAK* and *KLF12* in paraspinal muscle samples from AIS patients undergoing corrective surgery (*n* = 24).

**Figure 3:**
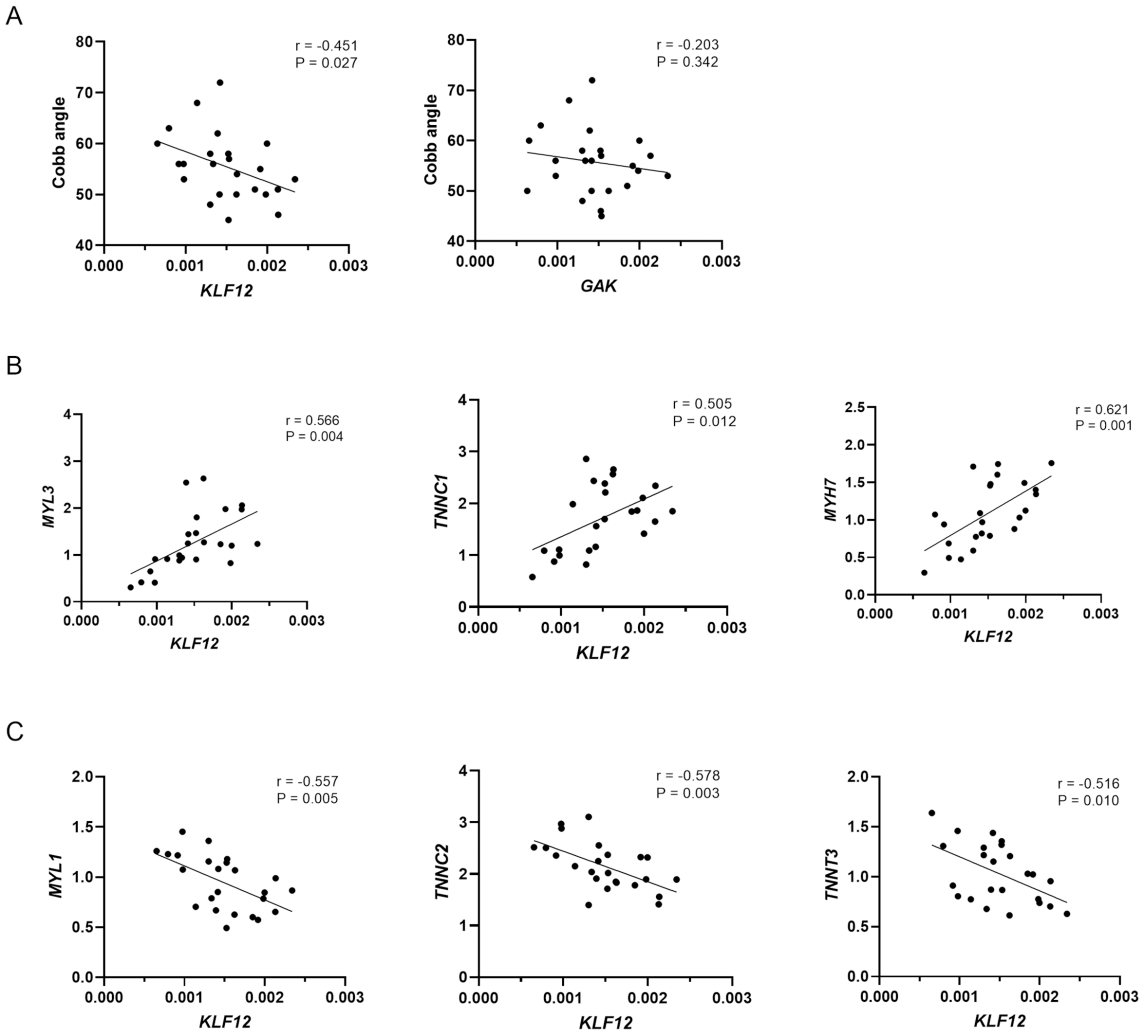
Relationship between expression levels of susceptibility genes and phenotypes in AIS. (A) Correlation between *KLF12* and *GAK* mRNA expression in paraspinal muscle tissue and Cobb angle in AIS patients (*n* = 24). (B) Correlation between *KLF12* expression and slow-twitch myofiber–associated genes (*MYL3, TNNC1*, and *MYH7*). (C) Correlation between *KLF12* expression and fast-twitch myofiber–associated genes (*MYL1, TNNC2*, and *TNNT3*).

As shown in Fig. 3A, *KLF12* mRNA levels were significantly negatively correlated with the Cobb angle (*r* = −0.451, *P* = 0.027), suggesting a potential association between lower *KLF12* expression and increased curve severity. In contrast, *GAK* expression showed no significant correlation with curve magnitude (*r* = −0.203, *P* = 0.342). Considering the critical role of muscle fiber composition in the pathogenesis of AIS, we further evaluated the relationship between *KLF12* expression, markers of slow and fast-twitch muscle fibers, and curve severity. As shown in Fig. 3B, *KLF12* expression was positively correlated with slow-twitch myofiber related genes, including *MYL3* (*r* = 0.566, *P* = 0.004)*, TNNC1* (*r* = 0.505, *P* = 0.012) and *MYH7* (*r* = 0.621, *P* = 0.001). In contrast, Fig. 3C demonstrated that *KLF12* expression was inversely correlated with fast-twitch myofiber–associated genes, such as *MYL1* (*r* = −0.557, *P* = 0.005), *TNNC2* (*r* = −0.578, *P* = 0.003), and *TNNT3* (*r* = −0.516, *P* = 0.010).

### Functional annotation of the susceptibility loci

Functional annotation of rs2061846 and rs7330031 revealed that both variants lie within intronic regions of *GAK* and *KLF12*, respectively, and map to genomic segments enriched for enhancer-associated histone modifications, including H3K36me3 and H3K4me1 (Fig. S2). Motif analysis further indicated that the allelic substitutions at these loci overlap predicted NKX2 and HMX2 transcription factor binding motifs. Consistent with these observations, genome browser inspection shows that both SNPs are positioned within putative regulatory elements characterized by accessible chromatin. As summarized in Table 4, SNPs in high linkage disequilibrium (*r*^2^ ≥ 0.9) within the same haplotype blocks were also predicted to have regulatory potential. These findings further support the involvement of these loci in transcriptional regulation processes potentially related to AIS development and progression.

**Table 4:** Functional annotation of novel variants associated with AIS severity.

**CHR**	**LD (r^2^)**	**Variant**	**MA**	**Enhancer histone marks**	**Proteins bound**	**Motifs changed**	**Selected eQTL**	**GENCODE genes**
4	0.93	rs17165087	T	MUS		CEBPB	29 hits	*GAK*
4	0.93	rs4690340	T	MUS		PPAR	30 hits	*GAK*
4	0.94	rs12509561	T			ATF3, Mxi1, RFX5	28 hits	*GAK*
4	0.95	rs2279175	A	MUS		7 altered motifs	29 hits	*GAK*
**4**	**1**	**rs2061846**	**C**	**MUS**		**Nkx2**	**29 hits**	***GAK***
4	0.95	rs56253935	T			13 altered motifs	29 hits	*GAK*
4	0.95	rs56307842	C			Ik-2, Mef2, STAT	29 hits	*GAK*
4	0.95	rs4690339	C	SPLN		4 altered motifs	30 hits	*GAK*
4	0.95	rs2335172	A	SPLN		Nkx2, Pax-6	29 hits	*GAK*
4	0.95	rs7684266	C				31 hits	*GAK*
4	0.95	rs3733353	A	4 tissues		4 altered motifs	31 hits	*GAK*
4	0.89	rs2306244	C	ESDR		5 altered motifs	28 hits	*GAK*
4	0.93	rs73207790	A	SKIN, MUS		15 altered motifs	26 hits	*GAK*
4	0.89	rs73207791	G	MUS		BHLHE40, Mxi1	26 hits	*GAK*
4	0.89	rs4690336	T	FAT, MUS	MAFK	TCF12	28 hits	*GAK*
4	0.81	rs60492656	T			8 altered motifs	29 hits	*GAK*
4	0.83	rs3775129	A			AP-2, CACD	28 hits	*GAK*
4	0.9	rs58429160	A			4 altered motifs	27 hits	*GAK*
4	0.8	rs17165130	A			6 altered motifs	28 hits	*GAK*
4	0.81	rs3736087	T	SKIN		5 altered motifs	30 hits	*GAK*
4	0.83	rs56785826	A	THYM, HRT, LNG		4 altered motifs	27 hits	*GAK*
4	0.82	rs4690204	T	SKIN, GI, PANC	POL24H8, SUZ12	5 altered motifs	27 hits	*GAK*
4	0.82	rs4690203	T	SKIN, GI, PANC	POL24H8, SUZ12	Hmx	22 hits	*GAK*
4	0.82	rs56223707	T	ADRL, SPLN		4 altered motifs	27 hits	*GAK*
4	0.81	rs3775127	A			BCL	30 hits	*GAK*
4	0.81	rs56080039	A			4 altered motifs	27 hits	*GAK*
4	0.8	rs4690202	C			Crx, Pitx2	23 hits	*GAK*
13	0.96	rs67074393	T			25 altered motifs		*KLF12*
13	0.96	rs7984021	T	LNG		5 altered motifs		*KLF12*
13	0.97	rs7990670	C			5 altered motifs		*KLF12*
13	0.97	rs17090405	T			6 altered motifs		*KLF12*
13	0.97	rs9573289	G			6 altered motifs	1 hit	*KLF12*
13	0.97	rs9565041	C			6 altered motifs		*KLF12*
**13**	**1**	**rs7330031**	**C**	**LIV**		**5 altered motifs**		***KLF12***

**Notes.**

Significantly associated loci identified in the validation are highlighted in bold.

CHR chromosome MA minor allele eQTL expression quantitative trait loci

## Discussion

Adolescent idiopathic scoliosis (AIS) is a complex spinal deformity with variable clinical outcomes. Multiple spinal components—including neuromuscular pathways, intervertebral disc (IVD) biology, and paraspinal muscle function—are reported to contribute to curve initiation and progression (Chan et al., 2023; Schlösser et al., 2014; Wang et al., 2024). These factors underline the heterogeneous nature of AIS and help explain the variability in clinical trajectories. For patients at high risk of curve progression, early implementation of preventive strategies such as bracing, core-strengthening exercises, and lifestyle modifications may help delay or prevent the development of severe curvature (Negrini et al., 2018). Although the genetic etiology of AIS has been extensively investigated through genome-wide association studies (GWAS) and whole-genome sequencing (Grauers, Einarsdottir & Gerdhem, 2016; Petrosyan et al., 2024), the contribution of genetic factors to curve progression remains poorly understood. Due to the limited number of identified susceptibility loci, accurate risk stratification at initial diagnosis remains challenging.

In this study, we identified two novel loci, rs7330031 (in *KLF12*) and rs2061846 (in *GAK*), that were significantly associated with AIS curve severity. The strongest association was observed for rs7330031, which is located within a region marked by active histone modifications and may alter a predicted HMX2 transcription factor binding motif, suggesting potential regulatory effects on *KLF12* expression. Further expression analysis revealed that *KLF12* mRNA levels in paraspinal muscles were significantly negatively correlated with the Cobb angle, suggesting a potential role in regulating curve progression. Moreover, *KLF12* expression was positively correlated with slow-twitch muscle fiber-related genes (*MYL3*, *TNNC1*, *MYH7*) and negatively correlated with fast-twitch fiber genes (*MYL1*, *TNNC2* and *TNNT3*). Interestingly, publicly available single-nucleus RNA-sequencing data from human skeletal muscle indicates that *KLF12* is expressed across several cellular compartments, including myofibers, fibroblasts and immune cells, with a markedly higher enrichment in slow twitch fibers (Kedlian et al., 2024). Because paraspinal muscle contains multiple cell types, the mRNA expression pattern of the whole tissue cannot fully capture fiber-type–specific alterations. Consequently, the association between *KLF12* downregulation and fiber-type signatures requires further experimental validation. Overall, these observations implicated the potential role for *KLF12* in regulating muscle fiber-type composition and contributing to AIS progression. This is consistent with previously reported AIS susceptibility genes, such as *LBX1*, which also implicate muscle-related pathways in curve development (Takahashi et al., 2011).

*KLF12* encodes a transcription factor of the Kruppel-like family and is known to repress *AP-2α* expression through direct promoter binding (Imhof et al., 1999). Notably, *AP-2α* was reported to inhibit skeletal myoblast proliferation by suppressing *FGFR1* activity (Mitchell & Di Mario, 2010). Abnormal growth of paraspinal muscle has been widely investigated in the etiology studies of scoliosis (Shahidi et al., 2021; Tam et al., 2022). Xu et al. (2021) reported that impaired myogenesis in the paraspinal muscle may predispose patients to curve development or progression. Taken together, our findings suggest that *KLF12* downregulation may contribute to AIS curve progression by disrupting myogenic signaling and altering muscle fiber composition.

The second significant SNP, rs2061846, maps in the intronic region of *GAK*. Functional annotation suggests that this variant may alter an Nkx2 transcription factor binding motif and potentially influence regulatory activity. However, unlike *KLF12*, *GAK* expression did not show a statistically significant correlation with the Cobb angle in our study (*r* = −0.203, *P* = 0.342). Although *GAK* encodes Cyclin G-associated kinase, a protein involved in intracellular trafficking and autophagy (Munson et al., 2021; Shimizu et al., 2009; Zhang et al., 2023). Notably, snRNA-seq resources indicate that *GAK* is enriched in macrophages, lymphocytes, and oligodendrocytes (Siletti et al., 2023), suggesting potential involvement in neural or immune pathways rather than muscle biology alone. Given the growing recognition of neuromuscular dysfunction as a contributing factor in scoliosis development (Koop, 2009; Krieger et al., 2011; Rummey et al., 2021), and a recent study has shown that variants affecting glycinergic neurotransmission can lead to abnormal spinal neural activity and scoliosis-like phenotypes (Wang et al., 2024), the possibility that *GAK* may influence curve progression through neural or neuroimmune mechanisms warrants further investigation.

In addition, although we evaluated AIS progression–associated variants reported in previous GWASs, none reached suggestive significance in our cohort. Such discrepancies may arise from differences in phenotype definitions, allele frequency distributions, or population-specific genetic architectures across studies. These observations underscore the need for replication in larger and more ethnically diverse cohorts to clarify the genetic basis of curve progression.

Several limitations of the present study should be acknowledged. First, patient selection was limited by incomplete treatment documentation and the lack of a mild-curve control group, which may introduce residual confounding in progression analysis. Future studies incorporating standardized treatment metadata and broader phenotype stratification will be needed to improve risk estimation. Second, due to ethical considerations, we could only collect paraspinal samples from patients with severe AIS undergoing corrective surgery, which limited our ability to assess gene expression across the full disease spectrum. Third, although associations between the identified SNPs and expression levels were observed, causal relationships have not yet been established. Future functional studies, such as CRISPR-based editing or reporter assays, are needed to validate the regulatory effects of the identified variants.

## Conclusions

Our study identified two novel susceptible loci associated with AIS curve severity. Among them, *KLF12* appears to be a particularly promising candidate, with both genetic association and expression data supporting its role in disease progression. These findings provide new insights into the genetic architecture of AIS progression and may contribute to the development of predictive biomarkers and targeted interventions for high-risk patients.

##  Supplemental Information

10.7717/peerj.20638/supp-1 Supplemental Information 1 QQ-Plot and PCA analysis

10.7717/peerj.20638/supp-2 Supplemental Information 2 Functional annotation of rs2061846 and rs7330031 (A) rs2061846 resides in an intronic region of *GAK* characterized by enhancer-associated histone modifications (H3K36me3, H3K4me1), predicted NKX2 transcription factor binding, and accessible chromatin signals. (B) rs7330031 is located within an intronic regulatory element of *KLF12*, enriched for PRDM6-associated marks and overlapping a predicted HMX2 transcription factor binding motif.

10.7717/peerj.20638/supp-3 Supplemental Information 3 Summary of previously reported AIS progression–associated SNPs evaluated in our discovery cohort

10.7717/peerj.20638/supp-4 Supplemental Information 4 RT-PCR primers

## Additional Information and Declarations

### Data Availability

The following information was supplied regarding data availability: The summarized GWAS outcome of the discovery cohort are available at Figshare: Dai, Zhicheng (2025). Summarized GWAS outcome. figshare. Dataset. https://doi.org/10.6084/m9.figshare.30022192.v1.
