# Supplementary figures and images for: Genome-wide association study identifies GAK and KLF12 associated with curve severity of adolescent idiopathic scoliosis
Source: PeerJ. 2026 Jan 19;14:e20638. doi: 10.7717/peerj.20638 (PMC12826036; doi:10.7717/peerj.20638)

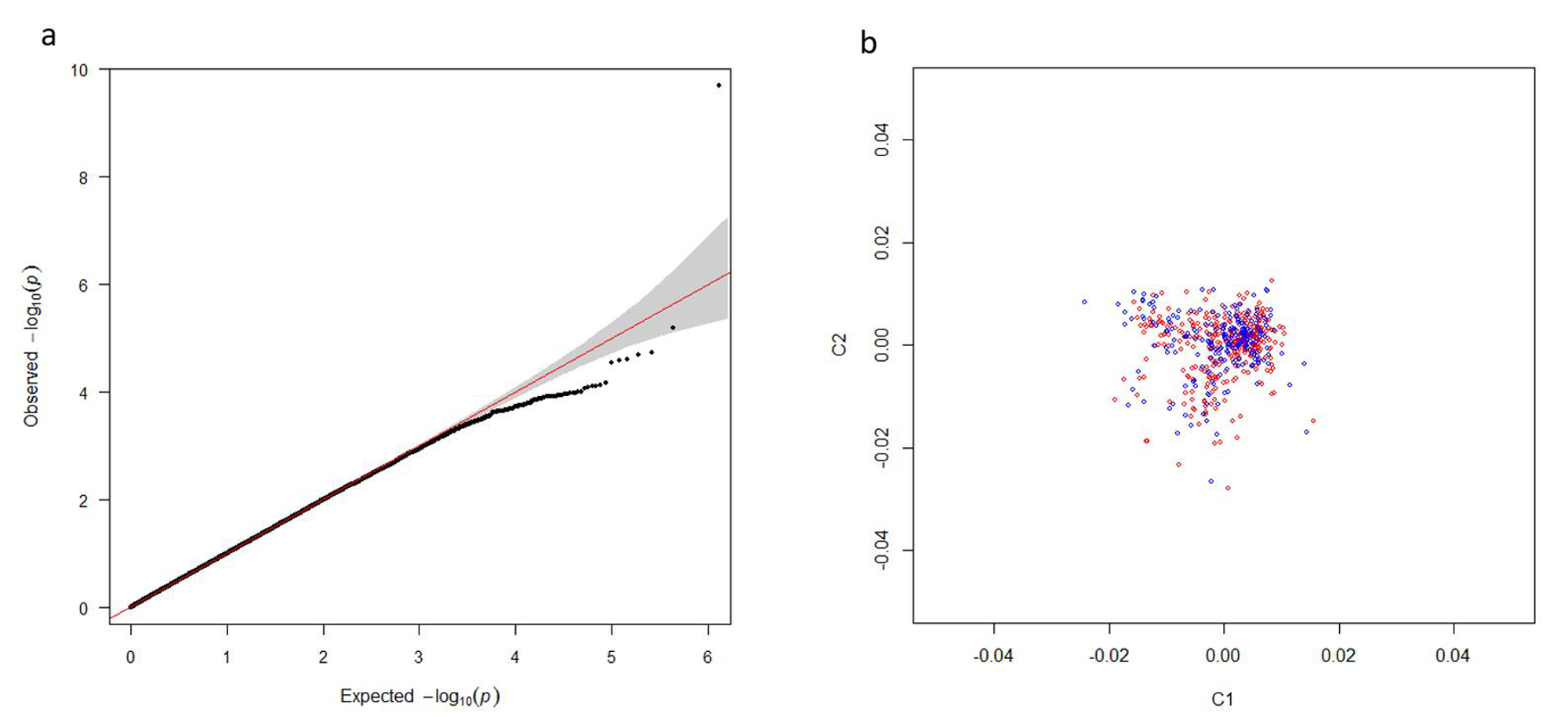

Supplement: Supplemental Information 1 [file peerj-14-20638-s001.png]

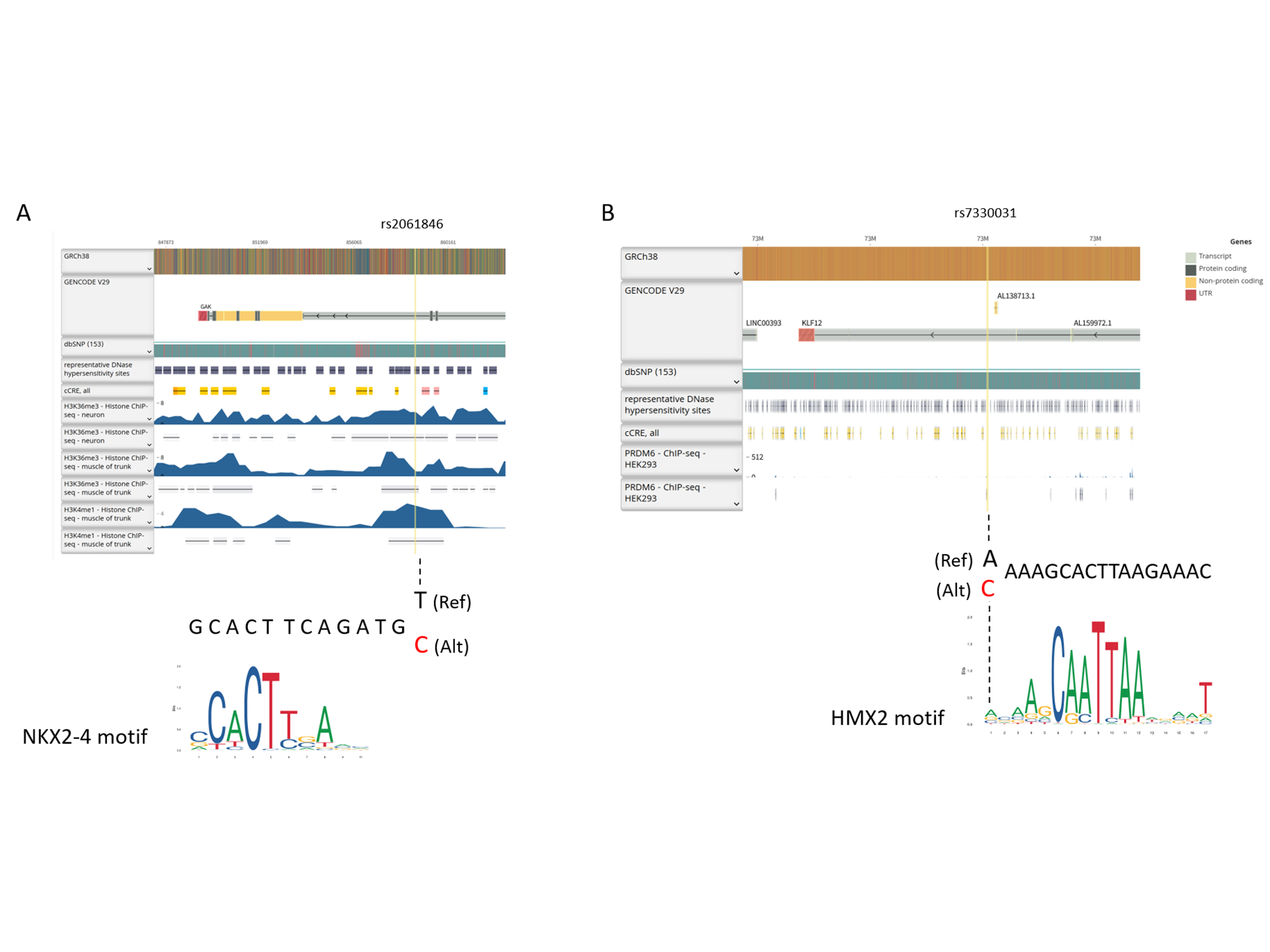

Supplement: Supplemental Information 2 — (A) rs2061846 resides in an intronic region of GAK characterized by enhancer-associated histone modifications (H3K36me3, H3K4me1), predicted NKX2 transcription factor binding, and accessible chromatin signals. (B) rs7330031 is located within an intronic regulatory element of KLF12, enriched for PRDM6-associated marks and overlapping a predicted HMX2 transcription factor binding motif. [file peerj-14-20638-s002.png]
