# Supplementary material for: Genome-wide association study identifies GAK and KLF12 associated with curve severity of adolescent idiopathic scoliosis
Source: PeerJ. 2026 Jan 19;14:e20638. doi: 10.7717/peerj.20638 (PMC12826036; doi:10.7717/peerj.20638)
Supplement: Supplemental Information 3 [file peerj-14-20638-s003.docx]

| SNP | Genes | CHR | Risk Allele | MAF | | P | OR |
| --- | --- | --- | --- | --- | --- | --- | --- |
|  |  |  |  | Severe  (n = 323) | Mild  (n = 297) |  |  |
| rs1828853 | MIR4300HG | 11 | - | - | - | - | - |
| rs10738445 | BNC2 | 9 | C | 0.4505 | 0.4801 | 0.3041 | 0.8875 |
| rs12946942 | SOX9/KCNJ2 | 17 | T | 0.2959 | 0.2741 | 0.41 | 1.113 |
| rs880633 | CHI3L1 | 1 | - | - | - | - | - |
| rs1538372 |  |  | - | - | - | - | - |
| rs4950881 |  |  | - | - | - | - | - |
| rs10399805 |  |  | - | - | - | - | - |
| rs6691378 |  |  | T | 0.3483 | 0.296 | 0.0538 | 1.271 |
| rs946261 |  |  | - | - | - | - | - |

**Supplementary Table 2 Summary of previously reported AIS progression–associated SNPs evaluated in our discovery cohort.**
