# Supplementary material for: Genome-wide association study identifies GAK and KLF12 associated with curve severity of adolescent idiopathic scoliosis
Source: PeerJ. 2026 Jan 19;14:e20638. doi: 10.7717/peerj.20638 (PMC12826036; doi:10.7717/peerj.20638)
Supplement: Supplemental Information 4 [file peerj-14-20638-s004.docx]

**Supplementary Table 1 RT-PCR primers**

| **Gene** | **Forward** | **Reverse** |
| --- | --- | --- |
| *KLF12* | CGGCAGTCAGAGTCAAAACAG | CGGCTTCCATATCGGGATAGT |
| *GAK* | CCACCCGAACATTGTCCAGTT | AGAACCGTGTCGCACGAAA |
| *MYL3* | TCACACCTGAGCAGATTGAAGA | GCTGGAGCATAGGCAGGAAAG |
| *TNNC1* | TGGTTCGGTGCATGAAGGAC | GTCGATGTAGCCATCAGCATT |
| *MYH7* | ACTGCCGAGACCGAGTATG | GCGATCCTTGAGGTTGTAGAGC |
| *MYL1* | GTTGAGGGTCTGCGTGTCTTT | ACCCAGGGTGGCTAGAACA |
| *TNNC2* | TGGGGACATCAGCGTCAAG | CCAAGAACTCCTCGAAGTCGAT |
| *TNNT3* | AGGAGCTGGTCGCTCTCAA | CCTTCTCTGCACGAATCCTCT |
| *GAPDH* | AGATCCCTCCAAAATCAAGTGG | GGCAGAGATGATGACCCTTTT |
